# Maternal-Effect Gene Expression in Cultured Preantral Follicles Derived from Vitrified-Warmed Mouse Ovary

**DOI:** 10.22074/cellj.2016.3742

**Published:** 2015-07-11

**Authors:** Roya Fatehi, Bita Ebrahimi

**Affiliations:** Department of Embryology at Reproductive Biomedicine Research Center, Royan Institute for Reproductive Biomedicine, ACECR, Tehran, Iran

**Keywords:** Vitrification, Ovary, Ovarian Follicle, Culture

## Abstract

**Objective:**

This study was conducted to assess survival of follicles, their oocyte maturation and fertilization potential as well as expression of early embryo developmental genes in *in vitro* cultured pre-antral follicles derived from vitrified-warmed mouse ovary.

**Materials and Methods:**

In this experimental study, ovaries of 12-day old Naval Medical Research Institute (NMRI) female mice were placed into non-vitrified and vitrifiedwarmed groups. Isolated preantral follicles from experimental groups were cultured in vitro for 12 days. On the 12^th^ day of culture, oocyte maturation was induced and then matured oocytes were *in vitro* fertilized. The rates of oocyte maturation and two-cell stage embryo formation were assessed. Relative expression of *Mater* and *Zar1* was evaluated on days 1, 6, 10 and 12 of culture. Data analysis was performed by t test and two-way ANOVA (P<0.05).

**Results:**

Our data showed no significant difference between the control and vitrification groups in the rate of follicular survival, oocyte maturation and two-cell stage embryo formation. The level of gene expression was higher on the 6^th^and 10^th^days of culture for *Mater* and *Zar1* in vitrified-warmed group compared with non-vitrified group, however, there was no significant difference between the two groups.

**Conclusion:**

It seems that the applied vitrification method did not reveal any negative effect on maturation and developmental competence of oocytes surrounded in preantral follicles and therefore could preserve follicular reserves efficiently.

## Introduction

Promotion of different therapeutic and diagnostic methods of oncology has led to an increase of survivors that have lost their fertility potential because of chemotherapy or radiotherapy. Ovarian tissue preservation, as an origin of oocytes and follicles in different stages, is a consequential matter for cancer-rescued females (1). Cryopreservation can be done by vitrification that seems to be a valuable method to preserve ovarian tissue (2) and is reported with high follicular survival rate in mammals (3, 4). A cryopreserved ovarian tissue can be used for transplantation (5) or follicle in vitro culture (IVC) (6) to achieve mature oocytes. Successful live births following grafting of cryopreserved ovarian tissue have been reported (7) but the risk of malignant-cell transfer in autotransplantation procedure or graft rejection in heterografting could not be ignored (8). Ovarian follicle IVC is an alternative option to avoid transplantation problems (6). IVC of isolated preantral follicles from fresh (9) and frozen-thawed mouse ovarian tissue has been successfully applied using two dimensional culture systems (10). Recent findings have shown murine oocytes from two dimensional culture systems are similar with *in vivo* developed oocytes in characteristics such as oocyte diameter, chromatin configuration, intracytoplasmic calcium signal transduction and meiosis capability (11). An efficient follicle culture procedure could produce a number of fertile competent oocytes (6) that could generate live offspring following *in vitro* fertilization (IVF) (12). This is not the only valuable point of IVC, it also provides the chance to study different aspects of follicle development such as oocyte-specific gene expression changes during this process (13). Accumulation of a large number of oocyte transcripts from primordial follicle to large antral stage is indicated in a microarray study of isolated mouse oocytes (14). Maternal-effect gene transcripts and proteins are expressed during oogenesis. They accumulate in the oocyte cytoplasm to work at the time of meiosis completion, mitosis initiation, embryonic genome activation and totipotential embryonic cell development (15). The first recognized oocyte-specific maternal-effect gene that plays important role at transition of oocyte to embryo in mice and humans was zygote arrest 1 (*Zar1*). It has been reported that expression of *Zar1* is restricted to ovary in mice (16). It has been shown that *Zar1*-null female mice are sterile and most embryos from *Zar1-/-* females are deprived to progress to the two-cell stage, *Zar1* is introduced as the first important identified gene that functions during oocyte to embryo transition. *Mater* is another maternal-effect gene and mouse embryos that lack its protein do not show normal embryonic genome activation, so it is necessary for early embryo development in mice (15). During late stages of folliculogenesis, when most transcripts are degraded, *Mater* is particularly transcribed and accumulated in oocytes which persists during embryogenesis. *Mater* and *Zar1* knockout models are infertile because of a block at the one- or two-cell stage embryo and this coincides with altered zygotic transcription (17). It has been shown that *Mater* and *Zar1* have a decreasing expression pattern during IVC of mouse preantral follicles (18). It has been shown that vitrification can affect gene expression; *Mater* and *Hook1* down-regulation and *Sod1* up-regulation was seen in mature mouse oocytes (19). In addition, decrease of *Gdf9* and *Bmp15* expression in sheep cumulus-oocyte complexes (COCs) following vitrification has also been reported (20).

According to the: i. probable changes of *Mater* and *Zar1* expression following ovary vitrification, ii. the important role of preantral follicle culture in embryo development and iii. the lack of knowledge in this regard, we decided to evaluate the effects of ovarian tissue vitrification on follicle survival and early embryo developmental gene expression in cultured preantral follicles. Also, *in vitro* oocyte maturation and fertilization were studied after ovary vitrification and follicle IVC to confirm the results of gene expression.

## Materials and Methods

### Study design

In this experimental study, ovaries were removed from 12-day old female mice (n=400) and distributed randomly into two experimental groups: non-vitrified control and needle immersed vitrification (NIV). All experiments were repeated 3 times for follicle IVC and gene expression. Non-vitrified control and vitrification groups were each divided into four subgroups according to the incubation time (i.e. days 1, 6, 10 and 12 of culture).

### Animals

Male and female adult Naval Medical Research Institute (NMRI) mice were purchased from Pasteur Institute of Iran and housed in rooms with controlled temperature (20-25˚C) and lighting (12 hour light: 12 hour dark). Animals were bred in the animal house of Royan Institute and provided with food and water ad libitum. They were handled according to the Ethical guidelines set by Royan Institute.

### Vitrification and warming

Isolated whole ovaries were first equilibrated in α-minimal essential medium (α-MEM, Gibco, Paisley, UK) supplemented with 7.5% ethylene glycol (EG, Sigma, MO, USA), 7.5% dimethyl sulphoxide (DMSO, Sigma, MO, USA) and 20% fetal bovine serum (FBS, Gibco, Paisley, UK) for 15 minutes at 4˚C in ice bath. They were then transferred into vitrification solution [α-MEM supplemented with 15% EG, 15% DMSO, 0.5 mol/L sucrose (Sigma, MO, USA) and 20% FBS] for 30 minutes at 4˚C. Afterwards, ovaries were first loaded by acupuncture needle (Dong Bang, Boryeong, Korea), then plunged in liquid nitrogen (LN_2_) and finally put in cryo-tubes (Grenier bioone, Germany) and stored in LN_2_ for a week.

For warming, vitrified ovaries were immediately immersed in warming solution I [α-MEM, 1 mol/L sucrose and 20% FBS] at room temperature for 10 minutes and then incubated in warming solution II [α-MEM and 10% FBS] at 37˚C for 30 minutes.

### Follicle in vitro culture and maturation

Non-vitrified fresh (control) and vitrifiedwarmed ovaries were put in 50 μl droplets of α-MEM with 10% FBS, and preantral (secondary) follicles with intact centrally-located oocyte and two or more layers of surrounding granulosa cells (110-130 μm diameter) were isolated mechanically using 29-G needle. They were then cultured individually in a 96-well plate (TPP, Switzerland) for 12 days. Culture medium was composed of α-MEM supplemented with 5% FBS, 5 mg/ml insulin, 5 mg/ml transferrin and 5 ng/ml sodium selenite (ITS, Gibco, Paisley, UK), 10 mIU/ml recombinant-follicle stimulating hormone (r-FSH, Merk, Germany) and 1 mIU/ml recombinant- luteinizing hormone (r-LH, Merk, Germany). It must be noted that r-LH was added only once at the beginning of the culture period. Follicles were cultured in 75 μl culture medium under 45 μl mineral oil and incubated at 37˚C 100% humidity and 5% CO_2_. Every 4 days, 30 μl of culture medium was replaced with fresh medium. Follicular survival rate was assessed after one, 6, 10 and 12 days of culture. Oocyte *in vitro* maturation (IVM) was induced on day 12 of culture by adding 1.5 IU/ ml human chorionic gonadotropin (HCG) and 5 ng/ml epidermal growth factor (EGF). After 16-18 hours, maturation was assessed under an inverted microscope by presence of the first polar body.

### In vitro fertilization

Sperm samples were collected from cauda epididymis of 6-8-week old male NMRI mice and capacitated in 500 μl droplets of T6 supplemented with 15 mg/ml bovine serum albumin (BSA, Gibco, Paisley, UK) at 37˚C, 100% humidity and 5% CO_2_ for 1 hour. For every three matured oocytes that was transferred to 50 μl IVF droplets composed of T6 and 15 mg/ml BSA, capacitated sperm was added to the droplets. These droplets were then kept at 37˚C 100% humidity and 5% CO_2_. After 4-6 hours, oocytes were washed to isolate attached sperms and pronuclei were examined under an inverted microscope to assess fertilization. Fertilized oocytes were transferred to development droplets composed of T6 and 4 mg/ml BSA at 37˚C, 100% humidity and 5% CO_2_. Two-cell embryo formation rate was assessed 24 hours later.

### Maternal-effect gene expression and RNA extraction

In order to evaluate gene expression, follicles in the control and vitrification groups were collected in three replicates (40 follicles in each replicate) after 1 day, 6 days (beginning of cumulus expansion), 10 days (antrum formation) and 12 days (antral follicle) of culture, pooled in Cell Reagent RNA Protect (Qiagen, Hilden, Germany) and stored at -70˚C until RNA extraction. Total RNA was extracted from each of the separate follicular pools (n=40) using an RNeasyMicro Kit (Qiagen, Hilden, Germany) according to the manufacturer’s instructions. Subsequently, cDNA was synthesized using RevertAid H Minus First Strand cDNA Synthesis Kit (Fermentas, Leon-Rot, Germany) and random hexamers according to the manufacturer’s instructions.

### Quantitative real-time polymerase chain reaction (PCR)

Specific primers for *Mater* and *Zar1* genes were designed by primer design software (AlleleIDPrimer Biosoft) (Table 1). The PCR mix was prepared according to a previous study (13). PCR was performed on Applied Biosystems Step One and Step One Plus Real time PCR systems according to a previous protocol (13). Reactions were performed in duplicate and the mean value of each duplicate was used for further calculations. "No template control" sample was run concurrently with test samples and a standard curve of amplification was developed using five serial dilutions of a reference cDNA (obtained from mouse ovarian tissue) for each gene. Relative quantification was calculated using Applied Biosystems’ software and the formula: Ratio=[E target]ΔCt target[control-sample]/[E ref]ΔCt ref[control-sample](13).

The quantification was normalized to an endogenous control (housekeeping gene *β-Tubulin*).

### Statistical analysis

Data of follicular survival, oocyte maturation, fertilization and two-cell embryo formation, and gene expression were analyzed by t test and two-way ANOVA respectively using SPSS software 17.0 (International Business Machines Corp, USA). Significance level was considered at P<0.05.

## Results

Follicles were considered healthy when their oocytes were clear, intact and more than 50% of their granulosa cells were viable. Follicular survival rate on different days of IVC procedure was lower in the vitrification group than the control group but these differences were not significant (Table 2). Also oocyte maturation and two-cell stage embryo formation rates were not significantly lower in vitrification group compared to the control one (Table 3).

**Fig.1:**
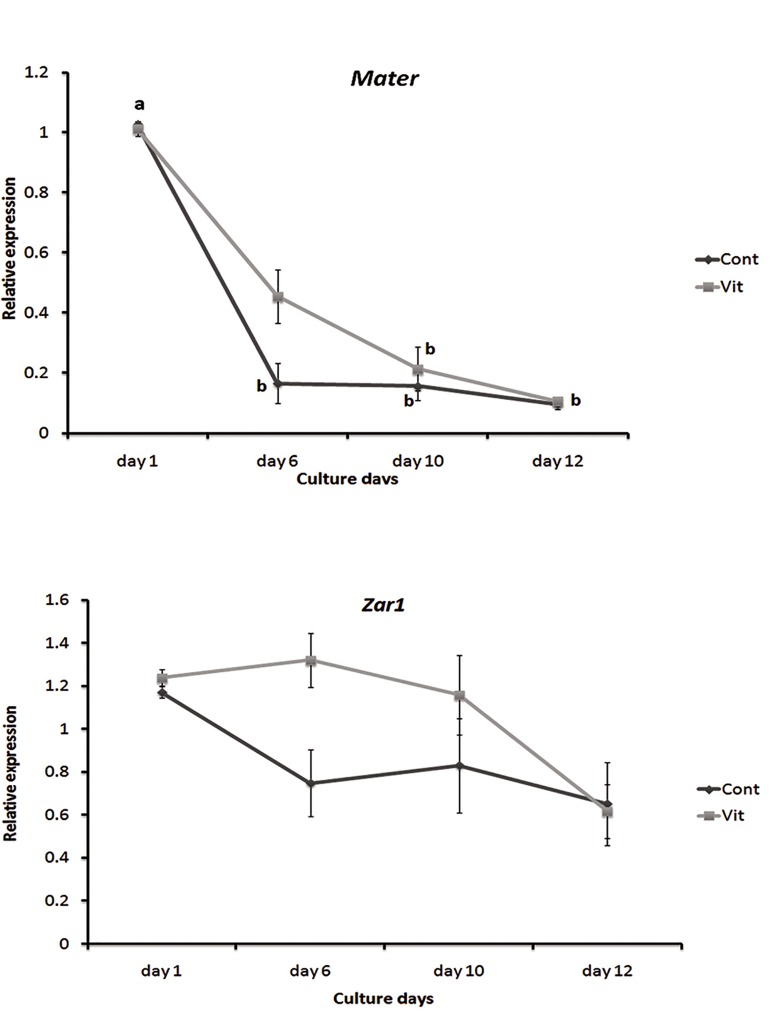
Relative expression of *Mater* and *Zar1* during different days of *in vitro* culture a vs. b shows significant differences (P<0.05).

Expression analysis of *Mater* and *Zar1* were performed in two series:

A analysis of gene expression pattern in each group during culture days. There was a decreasing pattern for *Mater* and *Zar1* during the 12 days of culture in both the vitrification and control groups. Comparison of *Mater* expression rate during culture days showed significant decrease between days 1 and 6 in the control group and also between day1 with days 10 and 12 in both groups (P<0.05). *Zar1* expression was not significantly changed in both groups on different days of culture.
B gene expression analysis of *Mater* and *Zar1* between groups in each certain day of culture period. *Mater* expression was increased non-significantly on the 6^th^ and 10^th^ days of culture in the vitrification group compared with the control one. Expression level of *Zar1* was almost the same on the first and 12^th^ days of culture in both groups but a non-significant increase was shown for this gene on days 6 and 10 in the vitrification group compared with the control group (Fig.1).

**Table 1:** Gene accession number, primer sequence, and product length


Genes	Accession number	Primer pair (5΄→3΄)		Product length (bp)

*Mater*	1.NM_001039143.1	F: CTGCGTTTCCAGTTCTTA		155
		R: AAGGGTTGTAGGATTTCTCA		
*Zar1*	1.NM_174877.3	F: GGATGATGTCTTGGCTTATG		154
		R: AGTTAGGATGTGTAGGTTGAA		
*Β-Tub*	NM_009371	F: GGAAGAGGATTTCGGAGAGG		78
		R: GGACAGAGGCAGCAGAAAG		


**Table 2:** Follicular survival rate during different days of in vitro culture in control and vitrification groups


Groups	Number of follicles	Healthy follicles			
		Day 1	Day 6	Day 10	Day 12

Control	130	100	95.33 ± 0.03	93.66 ± 0.01	91.33 ± 0.01
Vitrification	100	95.33 ± 0.03	89.33 ± 0.01	83.33 ± 0.04	82.33 ± 0.03


No significant difference was observed (P<0.05). Data were expressed as mean percentage ± standard error (SE). Each experiment was repeated 3 times. Statistical analysis was performed by t test.

**Table 3:** Maturation rate (mean percentage ± SE, 3 replicates) of in vitro cultured follicles derived oocytes in control and vitrification groups


Groups	Number of follicles	Healthy follicles			
		GV	GVBD	MII	Two-cell stageembryos

Control	115	7.33 ± 0.02	69.66 ± 0.02	22.33 ± 0.01	22.33 ± 0.01
Vitrification	80	10.33 ± 0.02	70 ± 0.03	20.66 ± 0.01	19.33 ± 0.01


No significant difference was observed (P<0.05). Data were expressed as mean percentage standard error (SE). Each experiment was repeated 3 times. Statistical analysis was performed by t test. GV; Germinal vesicle, GVBD; Germinal vesicle breakdown and MII; Metaphase II

## Discussion

Ovarian tissue cryopreservation is an efficient method for fertility preservation in reproductive medicine. Despite remarkable successes in applying this method, cryodamages on ovarian tissue and cells cannot be disregarded. The main purpose of this study was to evaluate the effects of ovarian tissue vitrification on follicle survival and early embryo developmental gene expression in cultured preantral follicles. Also, *in vitro* oocyte maturation and fertilization were studied after ovary vitrification and follicle IVC to confirm the results of gene expression. During the IVC procedure, different days were chosen for evaluation because of the following reasons: first day of culture for evaluation of the vitrification effects on follicle viability, day 6: beginning of cumulus cells expansion, day 10: time of antrum formation and day 12: antral follicle stage. No significant difference was observed in follicular survival between control and vitrification groups on the mentioned days. In this study, after 12 days of *in vitro* follicle culture, higher follicular survival was reported in both the control and vitrification groups (91.3 and 82.3% respectively) compared with previous studies (12, 21). *Mater* and *Zar1* are essential for early embryo development and are inherited by embryo through oocyte and it is why they are called maternal-effect genes (15, 17). This is the first study that investigates the expression pattern of *Mater* and *Zar1* during IVC of vitrified mouse ovarian tissue derived preantral follicles. Earlier studies demonstrated that vitrification (13, 20), IVC (13, 18) and IVM conditions (20, 22) could influence the expression pattern of a range of gene transcripts in ovine oocytes and murine follicles. Expression analysis of these genes in the present study did not reveal any significant difference between the control and vitrification groups during culture period. Previous reports have shown that the expression of *Mater* and *Zar1* (18) and other oocyte-specific gene (13) was decreased on the 12^th^ day compared to the initial days of culture. A decreasing expression pattern of *Mater* and *Zar1* during 12 days of culture in two experimental groups were similar to the mentioned studies (13, 21). Although a non-significant increase of gene expression was observed in the vitrification group compared with the control one during the middle days of culture, they reached the same level on day 12 of culture. It seems cryodamages that were shown on the 6^th^ and 10^th^ days of culture by gene expression enhancement could be compensated until day 12 of culture. To assess maturation and fertilization potential of IVC preantral follicles derived from vitrified ovarian tissue, oocyte IVM and IVF were also performed. As it shown in results, similar expression pattern of early embryo development genes in both experimental groups were confirmed by IVF outcomes in a way that the rate of two-cell embryo formation following IVC, IVM and IVF of preantral follicles were approximately the same in the control and vitrification groups. Lesser IVM and two-cell embryo formation rate were observed in this study in comparison with similar previous studies (23, 24). This could be correlated to the inappropriate concentration of used additive hormones in IVM media, incomplete cytoplasmic maturation of oocyte due to incapability of IVM media or failure to find the proper time for IVM induction and probability of oocyte post maturation. Therefore, to attain more favorable results, further studies regarding dosage optimization of hormones, improving IVM media and other factors seem to be needed.

## Conclusion

According to a non-significant difference of follicular survival, relative maternal gene expression, oocyte maturation and two-cell embryo formation rate between the control and vitrification groups, it seems that the applied vitrification method and IVC procedure did not reveal any negative effect on maturation and developmental competence of oocytes surrounded in preantral follicles and could thus be applied for mature oocyte enhancement.
